# The Tuberculin Skin Test (TST) Is Affected by Recent BCG Vaccination but Not by Exposure to Non-Tuberculosis Mycobacteria (NTM) during Early Life

**DOI:** 10.1371/journal.pone.0012287

**Published:** 2010-08-19

**Authors:** Sarah Burl, Uche J. Adetifa, Momodou Cox, Ebrima Touray, Hilton Whittle, Helen McShane, Sarah L. Rowland-Jones, Katie L. Flanagan

**Affiliations:** 1 Infant Immunology, Medical Research Council (UK) The Gambia, Fajara, The Gambia; 2 Nuffield Department of Clinical Medicine and the Jenner Institute, University of Oxford, Oxford, United Kingdom; 3 Weatherhall Institute of Molecular Medicine (WIMM), University of Oxford, Oxford, United Kingdom; Institute of Infectious Diseases and Molecular Medicine, South Africa

## Abstract

The tuberculin skin test (TST) is widely used in TB clinics to aid *Mycobacterium tuberculosis* (*M.tb*) diagnosis, but the definition and the significance of a positive test in very young children is still unclear. This study compared the TST in Gambian children at 4½ months of age who either received BCG vaccination at birth (Group 1) or were BCG naïve (Group 2) in order to examine the role of BCG vaccination and/or exposure to environmental mycobacteria in TST reactivity at this age. Nearly half of the BCG vaccinated children had a positive TST (≥5 mm) whereas all the BCG naïve children were non-reactive, confirming that recent BCG vaccination affects TST reactivity. The BCG naïve children demonstrated *in vitro* PPD responses in peripheral blood in the absence of TST reactivity, supporting exposure to and priming by environmental mycobacterial antigens. Group 2 were then vaccinated at 4½ months of age and a repeat TST was performed at 20–28 months of age. Positive reactivity (≥5 mm) was evident in 11.1% and 12.5% infants from Group 1 and Group 2 respectively suggesting that the timing of BCG vaccination had little effect by this age. We further assessed for immune correlates in peripheral blood at 4½ months of age. Mycobacterial specific IFNγ responses were greater in TST responders than in non-responders, although the size of induration did not correlate with IFNγ. However the IFNγ: IL-10 ratio positively correlated with TST induration suggesting that the relationship between PPD induced IFNγ and IL-10 in the peripheral blood may be important in controlling TST reactivity. Collectively these data provide further insights into how the TST is regulated in early life, and how a positive response might be interpreted.

## Introduction

The tuberculin skin test (TST) is used as a standard diagnostic tool to assess for infection with *Mycobacterium tuberculosis* (*M.tb*). Tuberculin purified protein derivative (PPD) is administered intradermally and the induration measured 48 – 72 hours later. It is generally accepted that in adults, a TST response ≥10 mm induration is indicative of *M.tb* infection, however in children the diagnostic ‘cut off’ is not so well defined.

There are a number of determinants that affect TST reactivity. The number of tuberculin units (T.U.) injected and the type of tuberculin can vary according to national guidelines [1]. Several reports suggest BCG vaccination at birth can result in TST reactivity ≥10 mm for 6 – 12 months [2], [3]. A longitudinal study in Taiwan of children vaccinated at birth suggested optimal ‘cut offs’ for children younger than 7 years of age should be >10 mm, with 21 mm being more appropriate for the first year of life [3]. Interestingly a TST induration between 5 and 10 mm can persist for up to 25 years after BCG vaccination [4]. This effect on TST reactivity is dependent on strain and dose of BCG used [5], [6], the method of vaccine administration [6], the time since vaccination [7], [8], the number of BCG vaccinations administered [9], the age, weight and nutritional status of the individual at the time of vaccination [8], [10] and genetic factors [11].

The TST can also be affected by cross reactivity with non-tuberculous mycobacteria (NTM) in the environment. A meta-analysis reported that in Montreal only 0.1% of TST responses ≥10 mm could be attributed to exposure to NTM compared to 2.3% in India where the prevalence of NTM is much greater [1]. Exposure to NTM has also been implicated in TST reactivity in Gambian BCG naïve infants whereby 22% (6/27) had a TST >5 mm at 4½ months of age [12].

There is little evidence to demonstrate that reactivity to the TST relates to protection against tuberculosis (TB), however delayed-type hypersensitivity (DTH) to mycobacterial antigens is associated with protection against leprosy [13]. Immune correlates of TST have not been well defined and there are conflicting reports on whether PPD induced peripheral blood responses correlate with the TST, making it difficult to interpret the diagnostic potential of the TST [12], [14].

This study aimed to examine how environmental mycobacterial exposure and BCG vaccination affect the TST in Gambian infants at 4½ months of age, comparing a population of infants that were BCG vaccinated at birth to a group that were BCG naïve at 4½ months of age. Our results suggest that BCG vaccination is likely to be responsible for TST reactivity at 4½ months of age, and that exposure to mycobacterial antigens in the environment had no effect on TST reactivity at this age. We also investigated the association between peripheral blood immune parameters and TST reactivity. We found that PPD induced IFNγ production in the peripheral blood correlated with TST reactivity, but not with TST induration, and the ratio of IFNγ: IL-10 positively correlated with the TST and therefore may be important in controlling TST reactivity.

## Methods

### Study design

**Figure 1:**
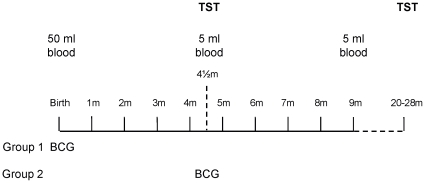
BCG vaccine schedule of study subjects. Babies were recruited at birth and randomised into either Group 1 (vaccinated at birth) or Group 2 (vaccinated at 4½ months of age) and followed up for 9 months. For all subjects a blood sample was collected at birth (50 mL cord blood), 4½- and 9- months of age (5 mL). A tuberculin skin test (TST) was performed at 4½ and at 20 – 28 months of age. Each child was followed up monthly for 9 months and then again at 20–28 months of age with a TB questionnaire (TBQ) and a general health questionnaire.

This study was approved by the Joint Gambia Government/MRC Ethics Committee and the London School of Hygiene and Tropical Medicine Ethics Committee as part of a larger longitudinal study. Gambian newborns were recruited at delivery from the Sukuta Hospital over a 2 year period. Following signed informed consent of a parent/guardian, newborns were randomised into one of two groups: Group 1 were vaccinated with BCG (0.05 mL intradermally, Serum Institute of India Ltd, Russian strain) at birth and Group 2 were vaccinated with BCG at 4½ months of age (Fig. 1). Exclusion criteria included low birth weight (<2.5 kg); twin pregnancy; intercurrent infection; exposure to TB at time of recruitment, or likelihood of moving location during the study period. All other childhood vaccines were administered according to the recommended Extended Programme on Immunisation (EPI) schedule in The Gambia (Table S1). Subjects were followed up in the first week of life, and then monthly to 9 months of age. Blood was collected at birth (50 mLs of cord blood) and at 4½- and 9-months of age (5 mL venous blood) into heparinised syringes (7.5 U/mL of blood) and transferred to the laboratories within 6 hrs of collection. This study describes immune responses related to TST reactivity at 4½ months of age; the more detailed longitudinal mycobacterial responses are described elsewhere [15]. At each visit a TB questionnaire was completed to assess for TB exposure. HIV status of the participants was not assessed but an estimated sero-prevalence of 2–4% among adults and 0.1% amongst children (0 – 14 years of age) in The Gambia [16] suggests that HIV was unlikely to be a significant confounder in this study. Recent studies in The Gambia have shown very low levels of helminth infection of between 0 – 3% in adult populations [17] suggesting helminth infection is also unlikely to be a confounding factor in the study.

### Tuberculin Skin Test (TST)

Reactivity to the TST was assessed at 4½ and 20–28 months of age in all subjects (Fig. 1). One dose of 0.1 mL of tuberculin PPD RT23 (containing 0.04 µg (2 Tuberculin Units), Statens Serum Institute (SSI), Denmark) was injected intradermally by a trained clinician or fieldworker. The induration (average of length and width) was measured 48 – 72 hours later and confirmed by a trained clinician. A positive response was considered to be ≥5 mm, and possible infection with *M.tb* if the TST was ≥10 mm. Any subject or close contact with a confirmed case of TB was referred to the TB clinic for further treatment. Infants found to be exposed to TB prior to BCG vaccination in Group 2 were immediately dropped from the study and monitored closely for TB disease.

### Cell culture conditions

From each blood sample, 500 µL of whole blood was cultured for 5 days with purified protein derivative of tuberculin (PPD, 25 µg/ml; SSI, Denmark), a positive control (Staphylococcal enterotoxin B, SEB, 5 µg/ml; Sigma Aldrich, UK) and an unstimulated negative control. After 24 hours in culture the whole blood was diluted 1∶5 with serum-free RPMI medium containing 2% penicillin/streptomycin (10,000 U/mL) and 1% L-glutamine, since optimization assays and previous studies [18] indicated better survival of cells in diluted blood. After 5 days of culture 100 µL of supernatant was collected and stored at −20°C to quantify the production of cytokines, and the remaining cells were phenotyped by flow cytometry.

### T cell Phenotyping

T cells were phenotyped according to manufacturer's instructions, fixed in 150 µL fix buffer (2% formalin in PBS) and processed on the 4 parameter BD FACS Calibur™ flow cytometer using the following antibodies: CD4 PE (SK3), CD4 APC (SK3), CD8 PerCP (SK1), CD25 FITC (M-A251), IL-10 PE (RM4-5), Ki-67 FITC (B56/MOPC-21) (BD Pharmingen, France) and FOXP3 APC (PCH101) (e-Bioscience, US). Antibody titrations and isotype controls were previously optimised in preliminary assays. Flow cytometric data was analysed using a template previously created during optimisations in Flow Jo software (Treestar, Oregon, US) as described previously [15] and as illustrated in Fig. S1, with minor sample specific modifications between experiments.

### Cytometric bead array

Supernatants were spun at 1500 rpm for 2 mins to pellet any precipitation in the sample. All samples stimulated with SEB were diluted 1∶2 with media containing 10% serum. A 6-plex system containing antibodies to IFNã, IL-10, IL-13, IL-6 and IL-17 (Th1 cytokine kit, Bio-Rad, California, US) was used to determine cytokine production using 50 µL of supernatant. The bead array assays were performed using 50 µL of sample according to manufacturer's instructions (Bio-Rad) and measured using the Bio-Plex 200 Suspension Array system (Bio-Rad). Standard curve outliers were eliminated by identifying samples where the coefficient of variance (%CV)>10% and observed/expected x 100 (obs/exp*100) was outside the range of 100 ^+^/− 20. Cytokine concentrations below the level of detection were calculated as zero in the analysis. All values that were greater than the highest range within the standard curve were repeated after further dilutions with media containing 10% human AB serum.

### Data collection and verification

Data points/samples were eliminated if any of the following problems arose: EPI vaccine administered less than 7 days prior to blood collection, cultures were contaminated, antibody staining was inadequate, the positive control (SEB) did not induce a response *in vitro* and acquisition of less than 1,000 lymphocytes. These issues occurred for approximately 10% of the >500 panels analysed, with 7% being due to a low number of lymphocytes, most frequently for the cord blood samples.

### Statistical analysis

For most values the data was not normally distributed and therefore non-parametric tests were used throughout. Cross sectional comparisons between groups were assessed using a 2-sided Mann-Whitney U test at 95% significance. * p = 0.009 – 0.050, ** p = 0.001 – 0.010, *** p<0.001. Correlations were analysed using Spearmans correlation coefficient. Bonferroni corrections were made according to the numbers of parameters assessed and the number of times a dataset was analysed. Data was analysed using R version 2.9.2 (The R Foundation, official part of the FSF's GNU project) and GraphPad Prism 5.01 (GraphPad Software Inc., US).

## Results

### Cohort Characteristics

103 neonates were recruited into the study at birth; 53 were randomised into Group 1 (vaccinated at birth) and 50 were randomised into Group 2 (vaccinated at 4½ months of age). Ninety infants were followed up at 4½ months (87.3%) and 85 at 20–28 months (82.5%). Details of this cohort are described elsewhere [15]; overall this study cohort was representative of the local Sukuta community [19].

### TST reactivity

**Figure 2:**
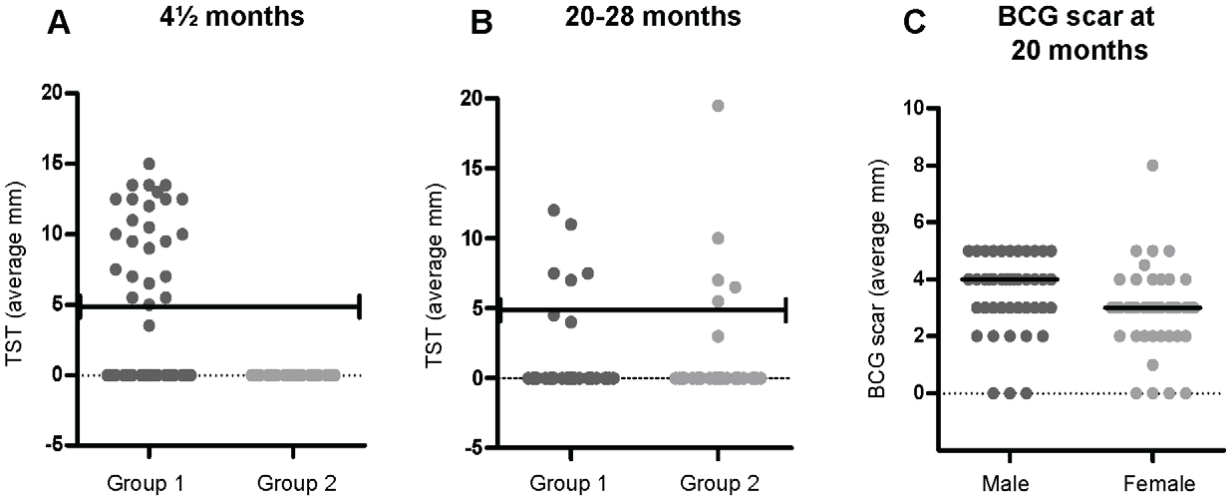
Tuberculin Skin Test (TST) and BCG scar results. Two T.U. PPD were injected into the forearm of each individual. The average length x width (mm) of the induration was measured 48 – 72 hours later at (A) 4½ months of age (Group 1, n = 51, Group 2, n = 39) and (B) 20– 28 months of age (Group1, n = 45, Group 2, n = 40). The black line represents a 5 mm ‘cut off’ for positive reactivity. (C) The relationship between scar and gender (male n = 42, female n = 43). Group 1 - vaccinated at birth, Group 2 - vaccinated at 4½ months of age.

At 4½ months of age all children received a TST. All BCG naïve subjects (Group 2) lacked reactivity to the TST (Fig. 2A). Among the BCG vaccinated group, 47% (24/51) of subjects exhibited a positive response (≥5 mm, responders or R) and the remaining 53% were non-responders (NR). Among the responders, 58% (14/24) had responses ≥10 mm and were therefore investigated for possible *M.tb* infection (Fig. 2A) but TB was not diagnosed in any of these individuals.

A repeat Mantoux test was undertaken on all subjects at 20 – 28 months of age. At this age all children had been vaccinated with BCG either at birth or at 4½ months of age. Only 10/85 (11.8%) children had a positive TST (≥5 mm) and 72/85 (84%) did not react to the TST at this later age. When comparing the TST results at 4½ and 20–28 months of age, it was clear that the timing of the BCG vaccination did not affect the TST at this later age, with similar numbers of positive subjects in both groups (Group 1: n = 5; Group 2: n = 5, p = 0.8868) (Fig. 2B). Four individuals had a response of ≥10 mm and were followed up for possible *M.tb* infection, but TB was not diagnosed in any of these individuals.

### BCG scar at 20 – 28 months of age

The presence and size of the BCG scar was recorded at the 20 –28 month visit and was found in 91.8% of individuals with a median diameter of 3 mm. Seven out of the eighty five (8.2%) children failed to develop a scar to BCG vaccination but reactivity to the TST was present in 30% of these (Table 1) suggesting that a lack of BCG scar was unlikely to be due to incorrect administration of the BCG vaccine. There was no relationship between lack of BCG scar and timing of BCG vaccination (4 children in Group 1 and 3 children in Group 2 failed to produce a scar).

**Table 1:** TST results and BCG scar formation at 20 – 28 months of age.

TST at 20–28 months of age (mm)	Number of individuals (% of total)	Number of individuals with scar (% within TST category)
0	72 (84.7)	67 (93.1)
1 – 4.5	3 (3.5)	2 (66.7)
5 – 9.9	6 (7.1)	5 (83.3)
≥10	4 (4.7)	4 (100)

There has been increasing interest in the role of gender in responses to vaccines [20], however there was no significant relationship between sex and reactivity to the TST at 4½ months of age (Fisher's Exact test p = 0.580) (Table 2). In addition, scarring after BCG vaccination was also not related to sex (data not shown) however, the median scar size was significantly larger in males (4 mm) than females (3 mm) (Fig. 2C) which has been shown previously in Malawian studies [21].

**Table 2:** The relationship between TST reactivity and gender at 4½ months of age.

		Female (n)	Male (n)
**Group 1**	**R**	12	12
**Group 1**	**NR**	16	11
**Group 2**	**NR**	19	20

R = responders ≥5 mm

NR = non responders<5 mm

### At 4½ months of age TST responders induce greater IFNγ production than non-responders in the peripheral blood

**Figure 3:**
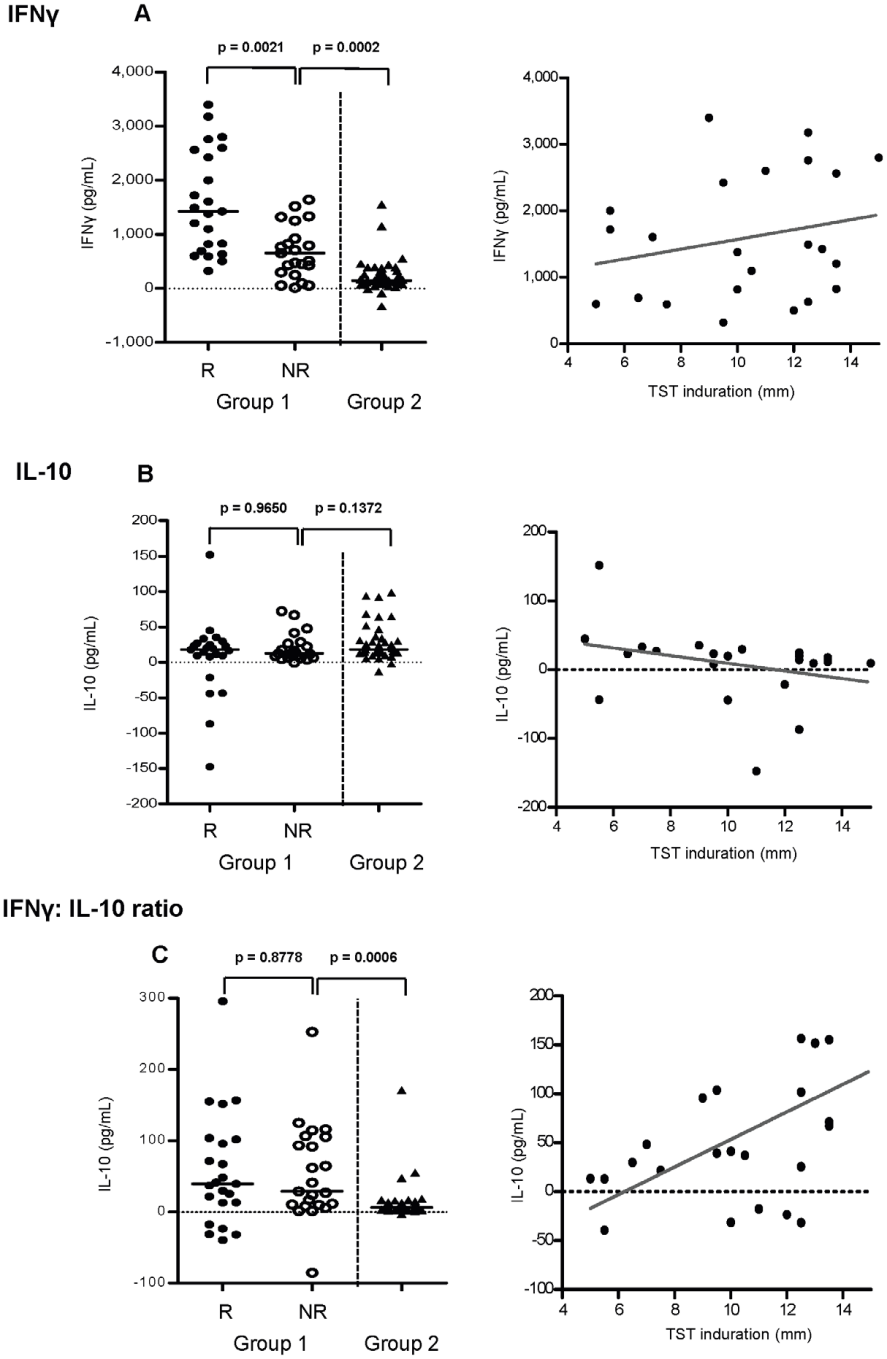
Relationship between TST and cytokine production. At 4½ months of age, whole blood was incubated with PPD for 5 days. (A) IFNγ and (B) IL-10 cytokine production and (C) the ratio of IFNγ: IL-10 was measured in the supernatants of the PPD cultures (left). Unstimulated control was subtracted from the PPD stimulated values, black bar represents the median value. A Mann-Whitney U test at 5% significance was applied to compare the cytokine production between groups (left graphs). Correlation between the TST induration of the responders and IFNγ, IL-10 and the ratio of IFNγ: IL-10 was calculated using Spearmans correlation coefficient at 5% significance (right graphs), dark grey line represents line of regression. R = responder n = 23, NR = non responder n = 22, unvaccinated n = 38.

The TST is caused by a localised delayed-type hypersensitivity (DTH) to PPD at the site of injection, but it remains unclear as to how TST reactivity relates to PPD responses in peripheral blood. At 4½ months of age the responders (R) within the BCG vaccinated group had higher *in vitro* IFNγ reactivity to PPD in peripheral blood than the non-responders (NR) (Fig. 3A). Interestingly, the NR in the vaccinated group, most of whom had an induration of zero, still exhibited greater IFNγ reactivity than the BCG naïve individuals from Group 2 that also had no reaction to the TST (Fig. 3A). Although R produced more IFNγ compared to NR, there was no correlation between the concentration of IFNγ produced and the size of induration of the TST reaction in R (r = 0.2317, p = 0.2875) (Fig. 3A). There was a borderline higher IL-6 production to PPD in responders (PPD; p = 0.0560) although this association was lost after correcting for multiple testing and did not correlate with TST induration. IL-13 and IL-17 were not significantly different between R and NR (IL-13 p = 0.6289 and IL-17 p = 0.7584) although they were significantly upregulated with BCG vaccination compared to the BCG naïve group at 4½ months of age (IL-13 p<0.0001, IL-17 p<0.0001, data not shown).

### IL-10 production in peripheral blood is inversely related to TST induration

At 4½ months of age IL-10 production in PPD culture supernatants was comparable between R and NR in the BCG vaccinated group (p = 0.9650, Fig. 3B). However, in those that responded to the TST, the quantity of IL-10 inversely correlated with the induration (r = −0.4396, p = 0.0358) (Fig. 3B). It was noticeable that the greater concentrations of IL-10 were in Group 2, although this was not significant when applying the Fisher's exact test or the Chi-square test for trend. Conversely, 83% of the individuals that downregulated IL-10 production (PPD stimulated IL-10 levels were lower than the unstimulated background) were TST responders, and this was significant using the Chi-square test for trend (p = 0.0485). BCG vaccinated infants had higher IFNγ: IL-10 ratios compared to unvaccinated infants (p<0.0001), but there was no significant difference between the R and NR (Fig. 3C). However there was a significant positive correlation between the IFNγ: IL-10 ratio and TST induration (Fig. 3C).

CD4^+^ and CD8^+^ T cell production of IL-10 (by ICS) in response to PPD did not differ between R and NR in Group 1, or Group 1 (R or NR) compared to Group 2 (data not shown). Furthermore, ICS IL-10 production did not correlate with TST induration, suggesting a non-T cell source for the IL-10 that correlates with TST induration.

### Lack of correlation between frequencies of nTregs and reactivity to the TST

**Figure 4:**
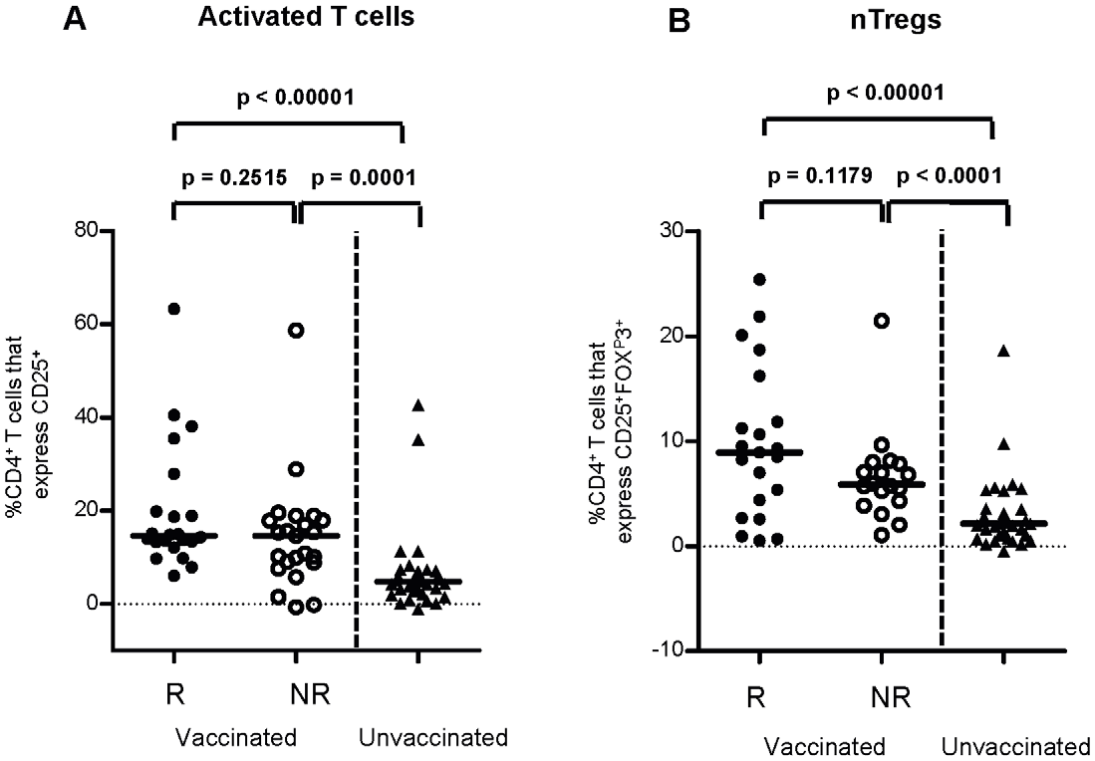
Relationship between TST and T cell phenotype in response to PPD. At 4½ months of age, whole blood was cultured with PPD for 5 days. (A) frequencies of activated T cells (CD4^+^CD25^+^) and (B) naturally occurring Tregs (CD4^+^CD25^+^FOXP3^+^) were measured with respect to TST reactivity. Unstimulated control was subtracted from the PPD stimulated values, black bar represents the median value. Comparisons between responders (R), non responders (NR) and unvaccinated individuals were calculated using a Mann-Whitney U test at 5% significance. R = responder n = 22, NR = non responder n = 21, unvaccinated n = 34.

Although IFNγ production in PPD cultures was greater in the R than the NR in group 1, activated T cells (CD4^+^CD25^+^) and naturally occurring Tregs (nTregs, CD4^+^CD25^+^FOXP3^+^) were comparable (Fig. 4A and B) suggesting that the TST reactivity is not related to these T cell populations. Similarly there was no relationship between TST and proliferating T cell frequencies (CD4^+^ Ki-67^+^ and CD8^+^ Ki-67^+^) (data not shown) in PPD cultures compared to the NR, although for all groups the frequencies were greater than the unvaccinated group (Fig. 4A and B).

## Discussion

TST reactivity in 4½ month old Gambian infants was found to be induced by BCG vaccination at birth. BCG naïve individuals responded to PPD *in vitro* suggesting mycobacterial exposure and priming, but lacked TST reactivity suggesting that the environmental mycobacterial exposure did not affect TST reactivity. Investigation of the immune correlates of TST reactivity in peripheral blood revealed greater IFNγ production in the TST responders but the absolute concentration of IFNγ did not correlate with induration. This discordance between IFNγ and TST induration indicates that the skin reaction and the size of the induration may be regulated by different factors. In support of this hypothesis Martins *et al* found strong erythema to the TST without induration which correlated with an increased IFNγ response to PPD [14]. IL-10 has been shown to decrease DTH in mice and strongly decrease expression of the skin homing receptor, cutaneous lymphocyte-associated antigen (CLA) on T cells [22], [23]. Our study found an inverse correlation between IL-10 and TST induration and a positive correlation between the ratio of IFNγ: IL-10 and TST reactivity suggesting IL-10 may contribute to regulating the size of the TST reaction. It would be interesting to examine the expression of CLA by T cells in the future in relation to TST reactivity. Regulatory T cells have also been implicated as another factor that controls delayed type hypersensitivity. Studies that have examined nickel allergy have found that functional Tregs reduce skin reactivity [24], however our results show no differences in the frequencies of nTregs between R and NR although Treg function was not examined. It is also worth noting that genetic factors may also contribute to how individuals respond to the TST [11], [25] but this was not examined in this study.

The *in vitro* studies showed strong PPD responses in the peripheral blood of the BCG naïve infants at 4½ months, possibly due to NTM exposure. However, none of these individuals reacted to the skin test at this age suggesting that environmental mycobacterial exposure does not affect the TST response at this age. This may be related to the mycobacterial dose or lack of cross reactivity between specific mycobacterial antigens in the environment and tuberculin PPD. Another explanation would be that the skin test is less sensitive than *in vitro* whole blood assays at detecting mycobacterial immunity. However previous work in The Gambia showed TST reactivity (≥5 mm) in 22% (6/27) of BCG naïve children at 4½ months of age [12]. Corrah *et al* has shown that prevalent NTM species differ between geographical locations within in The Gambia which could provide an explanation for this discrepancy between our findings and this latter study [26].

Not all individuals in the BCG vaccinated group responded to the TST, in fact 53% of these infants lacked any response to the TST (i.e. 0 mm induration). The possibility of false negative responses was considered. It has been shown that age (both the very young and the very old) can be responsible for false negative TST reactivity [27], [28] but other causes of false negative results include immunosuppression [29], overwhelming TB infection [30], other viral [29], [31] or bacterial infections [32] and recent viral vaccinations [33], [34]. In our study the TST and blood collection were only performed on healthy children and HIV rates in infants are very low in The Gambia suggesting the lack of TST reactivity observed in some individuals was not due to false negative responses.

The diagnostic use of the TST may be improved by modifying the ‘cut off’ values used to detect *M.tb* infection. Studies by Chan *et al* suggested that the ‘cut off’ in infants up to 1 year of age should be 21 mm, and that anything below this can be attributed to BCG vaccination [3]. Our study would support this as no child with TST reactivity >10 mm had any signs of *M.tb* infection or exposure to TB. By 20 months of age Chan proposed a ‘cut off’ of 18 mm in those vaccinated with BCG at birth [3]. Indeed 99% of children in our study had a TST<18 mm induration. Our data therefore supports using these higher ‘cut offs’ for suspected *M.tb* infection in young BCG vaccinated infants but future studies would be needed to verify this.

Overall our results showed that recent BCG vaccination affected TST reactivity in young infants but by 20 months of age the BCG vaccine had much less effect on the TST response. At 4½ months of age TST reactivity only occurred in the BCG vaccinated group, and those with responses >10 mm had no evidence of *M.tb* infection supporting the use of higher ‘cut offs’ for diagnosing *M.tb* infection or TB disease in this age group. Although the BCG naïve individuals did not react to the TST they responded to PPD *in vitro*, suggesting that environmental mycobacterial exposure did not prime a TST in this age group. Finally examining the immune correlates that relate to TST reactivity suggested that IFNγ correlated with TST reactivity but IL-10 inversely correlated with TST induration suggesting separate factors may be involved in controlling the TST reaction. These data therefore shed more light on how to interpret TST responses in young children who receive BCG vaccine early and are exposed to mycobacteria in the environment.

## Supporting Information

Table S1 Extended Programme of Immunisation (EPI) vaccine schedule in The Gambia (2006 – 2009). Apart from the changes in the BCG vaccine schedule illustrated in Figure 1, all other vaccines were administered according to the Extended Programme of Immunisation (EPI) in The Gambia.
(0.07 MB DOC)

Figure S1 Representative flow cytometry plots of PPD stimulated whole blood. 500 µL whole blood was cultured with PPD for 5 days. BCG vaccinated subjects (A and C) and unvaccinated (B and D) illustrating % CD25^+^ T cells gated on CD4^+^ T cells (A and B) and % CD25^+^FOXP3^+^ T cells gated on CD4^+^ T cells (C and D) (unstimulated samples on the left and PPD stimulated samples on the right).
(4.67 MB TIF)
