# Supplementary material for: The Tuberculin Skin Test (TST) Is Affected by Recent BCG Vaccination but Not by Exposure to Non-Tuberculosis Mycobacteria (NTM) during Early Life
Source: PLoS One. 2010 Aug 19;5(8):e12287. doi: 10.1371/journal.pone.0012287 (PMC2924396; doi:10.1371/journal.pone.0012287)
Supplement: Table S1 — Extended Programme of Immunisation (EPI) vaccine schedule in The Gambia (2006 – 2009). Apart from the changes in the BCG vaccine schedule illustrated in Figure 1, all other vaccines were administered according to the Extended Programme of Immunisation (EPI) in The Gambia. (0.07 MB DOC) [file pone.0012287.s001.doc]

**Supplemental Table 1: Extended Programme of Immunisation (EPI) vaccine schedule in The Gambia (2006 – 2009).**

| **At birth** | **1 month** | **2 months** | **3 months** | **4 months** | **9 months** | **>12 months** |
| --- | --- | --- | --- | --- | --- | --- |
| BCG |  |  |  |  |  |  |
| HepB |  | HepB |  | HepB |  |  |
| OPV | OPV | OPV | OPV |  | OPV | OPV (18 m) |
|  |  | DTwP | DTwP | DTwP |  | DTwP (16 m) |
|  |  | Hib | Hib | Hib |  | Hib (16 m) |
|  |  |  |  |  | MV |  |
|  |  |  |  |  | YF |  |

BCG = Bacillus Guerin Calmette

HepB = Hepatitis B vaccine

OPV = oral polio vaccine

DTwP = Diphtheria, tetanus, whole cell pertussis combined vaccine

Hib = Haemophilis influenza type B

MV = Measles vaccine

YF = Yellow fever vaccine

Apart from the changes in the BCG vaccine schedule illustrated in Figure 1, all other vaccines were administered according to the Extended Programme of Immunisation (EPI) in The Gambia.
