# Supplementary figures and images for: The Tuberculin Skin Test (TST) Is Affected by Recent BCG Vaccination but Not by Exposure to Non-Tuberculosis Mycobacteria (NTM) during Early Life
Source: PLoS One. 2010 Aug 19;5(8):e12287. doi: 10.1371/journal.pone.0012287 (PMC2924396; doi:10.1371/journal.pone.0012287)

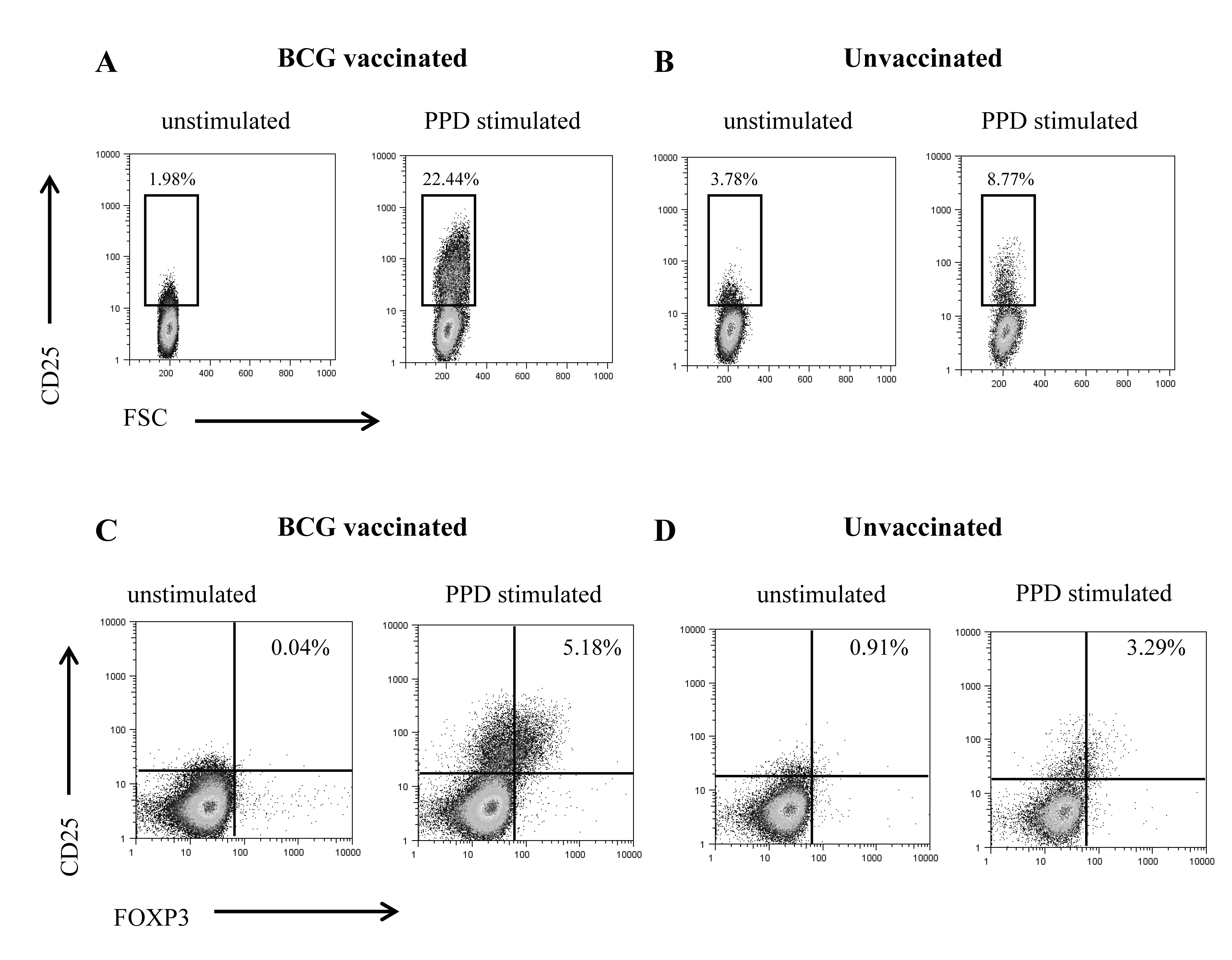

Supplement: Figure S1 — Representative flow cytometry plots of PPD stimulated whole blood. 500 µL whole blood was cultured with PPD for 5 days. BCG vaccinated subjects (A and C) and unvaccinated (B and D) illustrating % CD25+ T cells gated on CD4+ T cells (A and B) and % CD25+FOXP3+ T cells gated on CD4+ T cells (C and D) (unstimulated samples on the left and PPD stimulated samples on the right). (4.67 MB TIF) [file pone.0012287.s002.tif]
